# Effects of storage temperature on the change in size of *Calliphora vicina* larvae during preservation in 80% ethanol

**DOI:** 10.1007/s00414-012-0683-9

**Published:** 2012-03-08

**Authors:** Cameron S. Richards, Catherine C. Rowlinson, Martin J. R. Hall

**Affiliations:** Natural History Museum, Cromwell Road, London, UK

**Keywords:** *Calliphora vicina*, Forensic Entomology, Post-mortem interval, Preservation, Storage temperature

## Abstract

The size of immature blowflies is a common measure to estimate the minimum time between death and the discovery of a corpse, also known as the minimum post-mortem interval. This paper investigates the effects of preservation, in 80% ethanol, on the length and weight of first instar, second instar, feeding third instar, and post-feeding third instar *Calliphora vicina* larvae, at three different storage temperatures. For each larval stage, the length of larvae was recorded after 0 h, 3 h, 6 h, 9 h, 12 h, 24 h, 72 h, 7 days, 14 days, 30 days, 91 days, 182 days, 273 days, and 365 days of storage in 80% ethanol, at −25°C, 6°C and 24°C. Storage temperature had no statistically significant effect on the change in larval length and weight for all larval stages, but larval length and weight were significantly affected by the duration of preservation for first, second, and feeding third instar larvae, but not for post-feeding larvae. Generally, first and second instar larvae reduced in size over time, while feeding third instar larvae increased slightly in size, and post-feeding larvae did not change in size over time. The length of blowfly larvae preserved in 80% ethanol is not affected by constant storage temperatures between −25°C and +24°C, but we recommend that forensic entomologists should use the models provided to correct for changes in larval length that do become apparent over time.

## Introduction

Insects are commonly used to estimate the minimum time between death and the discovery of a corpse, also known as the minimum post-mortem interval (PMI_min_). The PMI_min_ is estimated by calculating the age of the oldest insect life stage i.e. eggs, larvae, pupae, collected from a body and/or crime scene. The larval stages of blowflies (Diptera: Calliphoridae) i.e. feeding first, second and third instars, and post-feeding third instar larvae are commonly found on corpses or at a crime scene, and make up the majority of entomological evidence [4]. Therefore, understanding the correct procedures for collecting, preserving and processing of this entomological evidence is essential when estimating a PMI_min_.

To calculate a comprehensive PMI_min_ estimate, a combination of preserved and live larvae is desirable. Larvae collected alive are reared through to their next developmental event at a known constant temperature, and the data are then modelled against precalculated developmental data to yield a PMI_min_ estimate. In addition, larvae are killed on collection, and preserved in 70–95% ethanol [2]. This method stops the development of the larvae at a known time, which reduces the potential error introduced into a PMI_min_ estimate when compared to those of live samples e.g. unknown temperature conditions of the larvae during transportation. The larval length, weight or width can be used as a measure of age to infer the PMI_min_ [6, 14]_._ Length is commonly the preferred measure as it offers a higher resolution than the weight or width [14].

Lord and Burger [12] was the first publication to mention adverse effects of killing methods and preservative solutions on immature and soft-bodied insects, but they supplied no supporting data or references to support this statement. The method of killing blowfly larvae and choice of preservative were first found to influence the length of larvae by Tantawi and Greenberg [17]. They tested the effects of 15 common preservatives on the length of feeding third instar and post-feeding larvae of *Protophormia terraenovae* and *Calliphora vicina*. They found that 70% ethanol had the least effect on larval length, although larvae reduced in size to result in an error of −7.2 h and −24.0 h for feeding third instar *C. vicina* and *P. terraenovae*, respectively, and −19.2 h and −9.7 h for post-feeding *C. vicina* and *P. terraenovae*, respectively, in a PMI_min_ estimate after 5 days of preservation. Finally, they noted that ‘maggots killed in boiling water, and then placed in preservative solutions, did not shrink’.

Adams and Hall [1] explored the effects of killing larvae in boiling water (termed ‘hot water killed’ (HWK)), before preservation, in more detail. They tested the change in length of post-feeding third instar *Lucilia sericata* and *C. vomitoria* larvae after HWK at 80°C and 100°C for 1, 30, 60 and 90 s, after storage in 80% ethanol and 10% formaldehyde solutions for differing durations, ranging from 3 h to 290 days. For the most reliable results, they recommended ‘larvae be killed by immersion in actively boiling water for not less than 30 s, measured immediately, and then transferred to 80% ethanol for preservation’. These recommendations have since been recognised as standard practice when collecting and preserving entomological evidence [2].

Day and Wallman [7] provided the most recent study to investigate the effects of preservation on the change in blowfly larval length. One of their experiments, relevant to this study, tested the effects of preservation on the change in length in 80% ethanol of first, second, feeding third and post-feeding third instar larvae of *Calliphora augur* over a 10-day period. Notable results included an average increase in length of 12.4%, 16.0%, 9.3% and 0.86% for first, second, feeding third and post-feeding third instar larvae, respectively, after a 10-day period.

Those are the only publications on the effects of killing methods and preservation on the change in size of blowfly larvae. Only one other study has looked at the effects of preservation on the change in larval size of forensically important insects, and that is Midgley and Villet (14) who used the beetle *Thanatophilus micans* (Coleoptera: Silphidae) as a test species. No study has looked at the effects of killing methods and preservation on the change in larval weight, as an alternative measure of size. Several studies have derived developmental data based on larval length and/or weight [3, 5, 8, 11, 13, 16], but some argue that weight is not as good as measure as length [10, 15, 19]. Although this may be true for larvae that have not been preserved, it is well known that the length of third instar larvae is significantly affected during preservation, even after 3 h [1]. Larval weight might not be as affected as larval length during preservation, and therefore, may prove to be a better measure of size than length.

Many other variables may influence the rate and degree of the change in larval size during preservation; temperature is one such variable. In a pilot study, Donovan et al. [8] noted that the length of post-feeding third instar *C. vomitoria* and *L. sericata* larvae was not affected during preservation in 80% ethanol at −20°C. This suggests that it might be possible to reduce or even negate the effects of preservation on the change in size of larvae using storage temperature.

This paper investigates the effects of preservation, in 80% ethanol, on the change in length and weight of first instar, second instar, feeding third instar, and post-feeding third instar *C. vicina* larvae, at three different storage temperatures. *C. vicina* is a Holarctic necrophagous blowfly species that is commonly found on animal and human remains. It is one of the most frequently encountered species in forensic investigations in the Holarctic region and is one of the few species that is present throughout the year in the United Kingdom [17].

## Materials and methods

Approximately 50 wild adult *Calliphora* flies were trapped alive, using a modified Red-top fly trap (Miller Methods, South Africa), in the wildlife garden of the Natural History Museum (51°29′46″ N:00°10′41″ W). These flies were then sexed but not identified to species, as this was more accurately achieved when the flies were killed and pinned. The females were placed each into a 25-ml plastic cup, covered by a 9-cm diameter Petri dish lid. Each pot contained a small piece of pork, approximately 2 cm^3^ in size, which served as an oviposition medium. Within 24 h, approximately half of the females laid eggs. These females were then numbered and killed, by placing them in a freezer for 30 mins. They were then pinned and identified to species. The egg batches were allowed to hatch at 23°C (±1°C), after which those of *C. vicina* were pooled.

Sixty newly hatched (between 0 h and 6 h old) *C. vicina* larvae were killed, by immersion in boiling water for no less than 30 s [1]. The larvae were dried on paper towel, after which their lengths were measured using a Dinolight Pro digital microscope and software, the weights were recorded using a Mettler AC100 microbalance, and their instars were recorded using a Wild M5 light microscope. (Note: Initial weights of first instar larvae were 0.0004 g but subsequent changes were less than the resolution of the microbalance (0.0001 g) and they were therefore not reweighed). They were then placed in 80% ethanol for preservation. Twenty larvae were stored at room temperature (mean 24.11°C, s.d. = 2.44°C, *n* = 52), twenty larvae were stored in a fridge (mean = 6.06°C, s.d. = 0.63°C, *n* = 2,048), and twenty larvae were stored in a freezer (mean = −25.81°C, s.d. = 2.28°C, *n* = 2,048). The above process was repeated on larvae aged 24 h, 48 h and 144 h. This meant that larvae were sampled at all developmental stages i.e. first instar, second instar, feeding third instar, and post-feeding third instar.

To test the effects of preservation on the change in size of larvae, the length and weight of each larva was re-recorded after 0 h, 3 h, 6 h, 9 h, 12 h, 24 h, 72 h, 7 days, 14 days, 30 days, 91 days, 182 days, 273 days, and 365 days. Specimens were processed quickly and the ethanol in all vials was topped up when necessary in an attempt to minimise any detrimental effect of handling specimens during preservation [7]. The variation between persons recording this data was assessed prior to the experiment and was concluded to be negligible (results not shown).

### Data analysis

The data were captured in ‘Microsoft Excel 2003’ and analysed in a two-way ANOVA and a Tukey post hoc test in the computer software package ‘Statistica 9’.

Adams and Hall [1] found that the length of larvae was most significantly affected within the first 9 h of preservation. In order to analyse and present the results of these early stages of preservation in more detail, data including 0 h, 3 h, 6 h, 9 h, 12 h, 24 h, and 72 h samples were analysed together, termed ‘short-term storage’, while data including 0 h, 3 days, 7 days, 14 days, 28 days, 91 days, 182 days, 273 days, and 365 days were analysed together and termed ‘long-term storage’. This limited the maximum temporal samples of any analysis to nine, enabling clarity in graphical presentation of the detail of variation within and between data sets.

Because the time interval of the sampling strategy ranged hugely i.e. from hours to 1 year, time was plotted as a categorical variable on the x-axis rather than as a continuous variable. This spread the data evenly, making it possible to observe any trends.

### Implication for estimating PMI_min_

To test the effect of maximum change in larval length during preservation on a PMI_min_ estimate using blowfly larvae, the percentage error between hypothetical PMIs_min_ calculated from both the original lengths (at time zero) and the maximum change in larval lengths, regardless of storage temperature, was calculated. The minimum, mean and maximum larval lengths from this study were used to calculate PMI_min_ for each instar for two example temperatures (10°C and 20°C).

## Results

Generally, first and second instar larvae reduced in size during both short-term and long-term storage in 80% ethanol, while feeding third instar larvae increased slightly in size, and post-feeding larvae did not change in size.

### Effects of storage temperature and time on the change in larval weight during preservation

There was no significant difference in the change in weight over time between storage temperatures for all larval ages (*p* > 0.13 < 1.00). But, storage time had a significant effect on the change in weight of second (short-term storage (*F* (6, 395) = 26.07, *p* < 0.00); long-term storage (*F* (8, 484) = 87.74, *p* < 0.00), and post-feeding third (short-term storage *F* (6, 393) = 2.88, *p* < 0.01); long-term storage (*F* (8, 505) = 3.38, *p* < 0.00) instar larvae. Storage time did not have a significant effect on the change in weight of feeding third instar larvae for short-term (*F* (6, 399) = 1.98, *p* > 0.07) or long-term (*F* (8, 485) = 1.83, *p* > 0.07) storage.

**Fig. 1:**
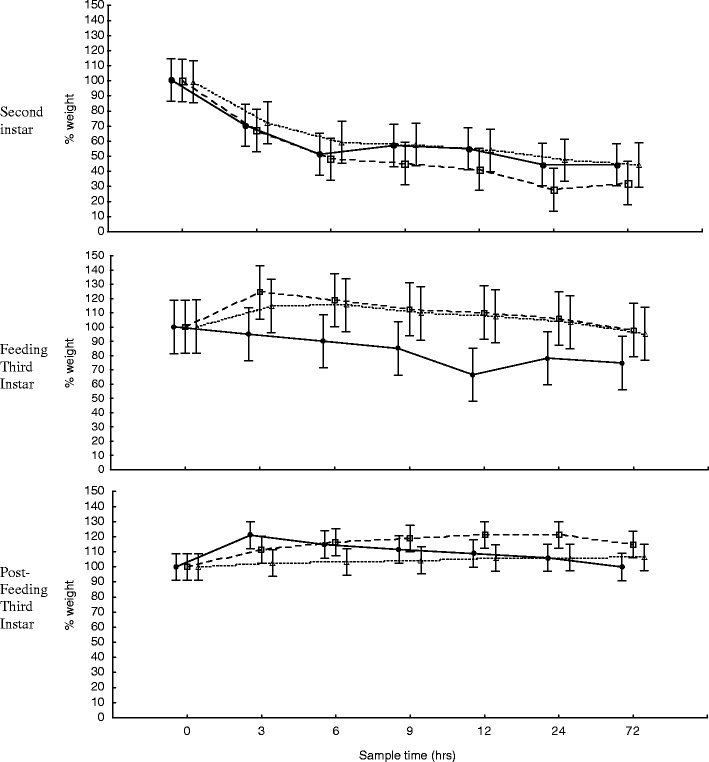
Change in larval weight for the first 72 h of storage at −25°C (*unfilled triangle*), 6°C (*unfilled square*), and 24°C (*filled circle*) of second instar (mean weight = 0.0049 g at time zero, *n* = 20), feeding third instar (mean weight = 0.0147 g at time zero, *n* = 20), and post-feeding third instar (mean weight = 0.1037 g at time zero, *n* = 20) larvae. *Error bars* represent ±95% confidence intervals

**Fig. 2:**
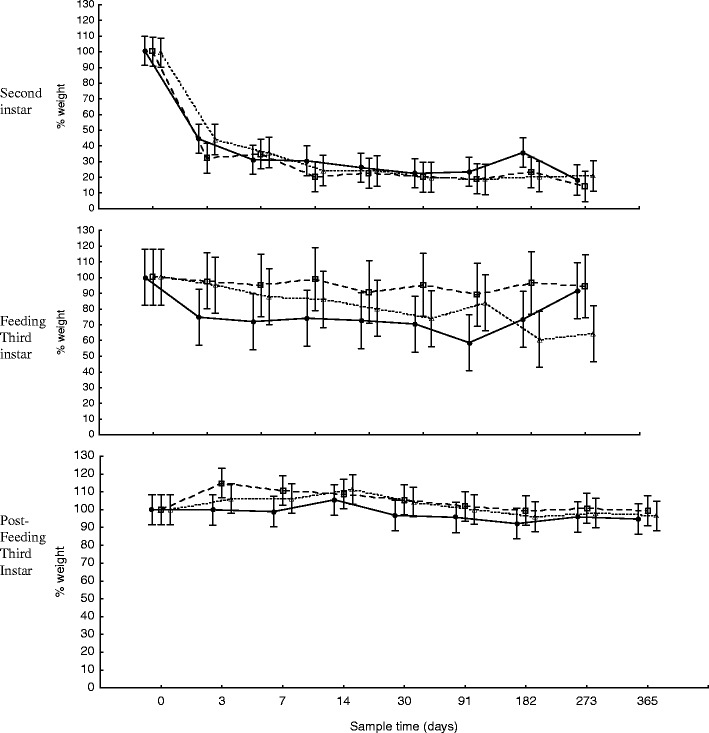
Change in larval weight for the 365 days of storage at −25°C (*unfilled triangle*), 6°C (*unfilled square*), and 24°C (*filled circle*) of second instar (mean weight = 0.0049 g at time zero, *n* = 20), feeding third instar (mean weight = 0.0147 g at time zero, *n* = 20), and post-feeding third instar (mean weight = 0.1037 g at time zero, *n* = 20) larvae. *Error bars* represent ±95% confidence intervals

**Fig. 3:**
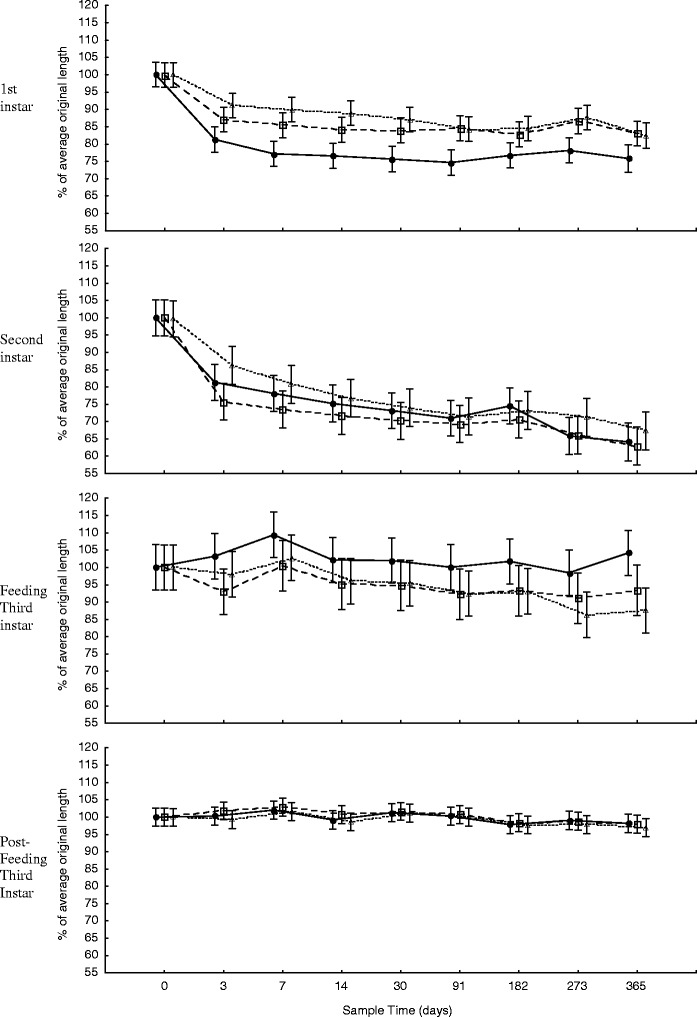
Change in larval length for the 365 days of storage at −25°C (*unfilled triangle*), 6°C (*unfilled square*), and 24°C (*filled diamond*), for first instar (mean length = 3.36 mm at time zero, *n* = 20), second instar (mean length = 6.55 mm at time zero, *n* = 20), feeding third instar (mean length = 9.58 mm at time zero, *n* = 20), and post-feeding third instar (mean length = 17.58 mm at time zero, *n* = 20) larvae. *Error bars* represent ±95% confidence intervals

**Fig. 4:**
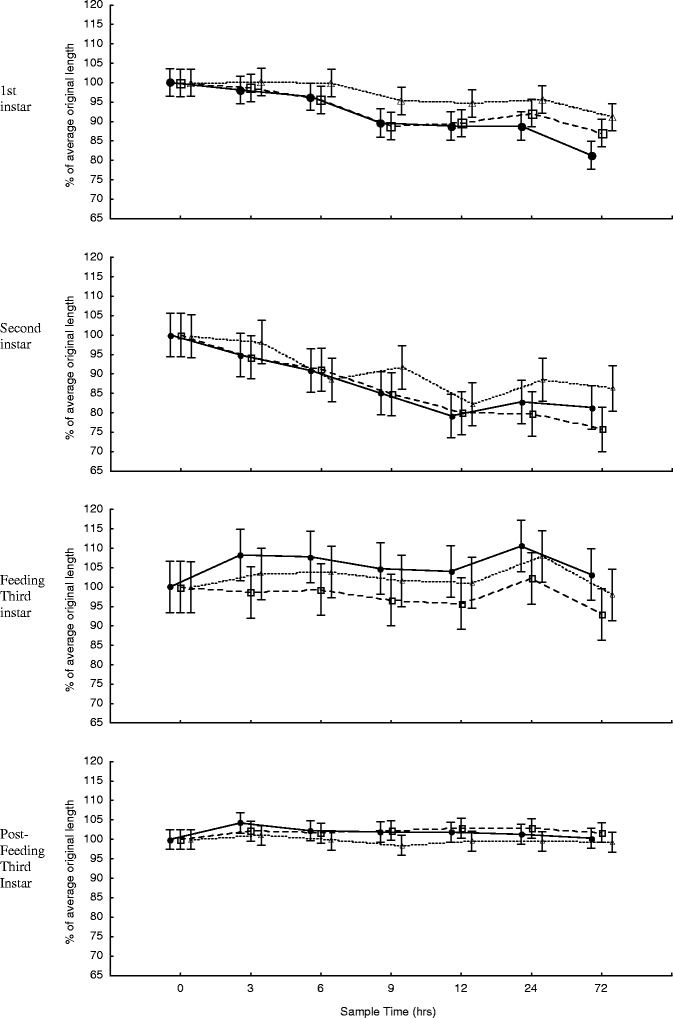
Change in larval length for the first 72 h of storage at −25°C (*unfilled triangle*), 6°C (*unfilled square*), and 24°C (*filled circle*), for first instar (mean length = 3.36 mm at time zero, *n* = 20), second instar (mean length = 6.55 mm at time zero, *n* = 20), feeding third instar (mean length = 9.58 mm at time zero, *n* = 20), and post-feeding third instar (mean length = 17.58 mm at time zero, *n* = 20) larvae. *Error bars* represent ±95% confidence intervals

Larvae lost a greater percentage of weight than length during both short-term and long-term storage (compare Figs. 1 and 2 (weight) to Figs. 3 and 4 (length)). Second instar larvae weighed an average of 40.3% (s.e. = 2.7%) and 17.8% (s.e. = 1.3%) of their original weight after short-term (3 days) and long-term (365 days) preservation, respectively, for all storage temperatures. Feeding and post-feeding larvae weighed an average of 89.3% (s.e. = 4.4%) and 82.7% (s.e. = 5.5%), and 107.2% (s.e. = 2.6%) and 96.9% (s.e. = 2.2%) after the same durations, respectively. The greatest change in weight for second instar larvae was a decline to 14.1% of their original weight after 365 days at fridge temperature. The greatest change in weight for feeding and post-feeding third instar larvae was a decline to 58.6% and a rise to 121.0% after 182 days (6 months) and 3 h at room temperature, respectively.

### Effects of storage temperature and time on the change in larval length during preservation

During both short-term and long-term storage, the change in length was consistently least, i.e. closest to 100% of the original length, with samples that were stored in the freezer when compared to those stored in the fridge or at room temperature for all larval instars (Figs. 3 and 4), but this was not statistically significant, i.e. there was no significant difference in the change in length over time between storage temperatures for all larval ages (*p* > 0.12 < 1.00). However, storage time had a significant effect on the change in length of first (*F* (6, 395) = 22.59, *p* < 0.00) (*F* (8, 495) = 32.68, *p* < 0.00), second (*F* (6, 395) = 20.37, *p* < 0.00) (*F* (8, 484) = 44.35, *p* < 0.00), and feeding third (*F* (6, 399) = 2.29, *p* < 0.04) (*F* (8, 485) = 3.06, *p* < 0.00) instar larvae, for short-term and long-term storage, respectively. Storage time also had a significant effect on the change in length of post-feeding third instar larvae during long-term storage (*F* (8, 505) = 3.9, *p* < 0.00) but not for short-term storage (*F* (6, 393) = 1.1, *p* < 0.38).

The greatest changes in length for first and second instar larvae were shrinkages recorded after 365 days of storage, when they measured 75.8% (s.e. = 1.8%) of the average original length (i.e. at time 0 h) at room temperature and 62.7% (s.e. = 1.9%) of the average original length at fridge temperature, respectively (Fig. 3). The greatest changes in length for feeding and post-feeding third instar larvae were lengthening recorded at 110.5% (s.e. = 2.7%) and 104.8% (s.e. = 0.7%) of the original length, after 24 h at room temperature and 3 h at room temperature, respectively (Fig. 4). The average variation over time, expressed in terms of standard error, was 1.8%, 2.4%, 3.4% and 1.3% of the mean length for first, second, feeding third and post-feeding third instar, respectively for all storage temperatures combined.

### Implication for estimating PMI_min_

The percentage errors in PMI_min_ calculated from length after preservation were greatest for second instar larvae, reaching a maximum of −53.0%, and least for post-feeding third instar larvae, reaching a maximum of only +5.3%. The percentage errors in PMI_min_ calculated from the minimum lengths were consistently larger than those calculated from the mean and maximum values within each instar, and these differences were greatest for first instar larvae, differing as much as 12% between minimum and maximum values, and smallest for post-feeding third instar larvae, differing as little as 0.1% between minimum and maximum values. The percentage errors in PMI_min_ were consistently larger than the percentage change in original length for all larval lengths within all larval instars (Table 1).

**Table 1:** The differences between estimates of the hypothetical PMI (in hours) based on the original larval length and on the maximum changes in larval length due to preservation (adjusted length) for first, second, feeding third and post-feeding third instar larvae

Instar	Summary statistic	Original length (mm)	Adjusted length (mm)	Adjusted length as a percent of original length	PMI_min_ (h) at 10°C based on:		PMI_min_ (h) at 20°C based on:		Percent error in PMI_min_ when using adjusted length
					Original length	Adjusted length	Original length	Adjusted length	
First	Minimum	2.7	2.0	75.8^a^	19.6	9.9 (9.7)	9.3	4.7 (4.6)	−49.5
	Mean	3.4	2.6	75.8^a^	29.9	17.7 (12.2)	14.2	8.4 (5.8)	−40.7
	Maximum	3.9	3.0	75.8^a^	37.3	23.4 (14.0)	17.7	11.1 (6.6)	−37.5
Second	Minimum	4.6	2.9	62.9^b^	47.7	22.4 (25.3)	22.6	10.6 (12.0)	−53.0
	Mean	6.5	4.1	62.9^b^	75.9	40.1 (35.7)	35.9	19.0 (16.9)	−47.1
	Maximum	8.0	5.0	62.9^b^	98.1	54.1 (44.0)	46.5	25.6 (20.8)	−44.8
Third feeding	Minimum	6.3	5.4	86.3^c^	72.9	60.1 (12.8)	34.5	28.5 (6.1)	−17.5
	Mean	8.6	7.4	86.3^c^	107.0	89.5 (17.5)	50.7	42.4 (8.3)	−16.3
	Maximum	12.5	10.8	86.3^c^	164.7	139.4 (25.4)	78.0	66.0 (12.0)	−15.4
Third post-feeding	Minimum	14.7	15.4	104.8^d^	197.3	207.8 (10.5)	93.5	98.4 (5.0)	+5.3
	Mean	17.6	18.4	104.8^d^	240.3	252.8 (12.5)	113.8	119.8 (5.9)	+5.2
	Maximum	19.3	20.2	104.8^d^	265.5	279.2 (13.7)	125.8	132.3 (6.5)	+5.2

The difference was calculated from the original mean length and from the maximum amount of change in mean length during preservation, regardless of storage temperature, at two different constant developmental temperatures (10°C and 20°C). Thermal summation constants acquired from Donovan et al. (2006) (Lower developmental threshold = 1°C). Data in brackets is the difference in hours between the PMI_min_ before and after storage

^a^Maximum post preservation change based on 24°C after 365 days

^b^Maximum post preservation change based on +4°C after 365 days

^c^Maximum post preservation change based on −25°C after 273 days

^d^Maximum post preservation change based on +24°C after 3 h

## Discussion

### Effects of storage temperature and time on the change in larval weight during preservation

The change in larval weight was consistently greater than the change in larval length, at all storage temperatures, over the same time periods. This is particularly true for second instar larvae, which lost 47.0% more average weight than average length after 365 days of storage. The range in the change in larval weight at any given sample time was much greater than the range in the change in larval length for the same time period. These data show that weight is far more susceptible to change than length during preservation. This sensitivity to preservation in 80% ethanol is not desirable in forensic entomology, as such changes in size, if not taken into account, can have a direct influence on a resultant PMI_min_ estimate.

An important effect of large range variation within the weight data set is that it yielded large confidence intervals (Figs. 1 and 2) resulting in non-significant interactions which limited the forensic value of the data. A much larger sample size (*n* > 100) would be needed to derive any reliable significant difference in these data. This level of variation at least partly explains why no significant differences were found between the changes in weight of larvae stored at different temperatures, despite the trends between storage temperatures of feeding third instar larvae for both short-term and long-term storage being quite different from one another (Figs. 1 and 2). In comparison, the trends for change in larval weight at different storage temperatures were very similar for second instar and post-feeding third instar larvae.

Our data demonstrate that larval weight is an unreliable measure of age for larvae that have been stored in 80% ethanol, due to the large variability in the change in larval weight during preservation. Larval length was a more reliable alternative.

### Effects of storage temperature and time on the change in larval length during preservation

Storage temperature had no statistically significant effect on the change in larval length during preservation in 80% ethanol over time. However, the larvae stored at −25°C were consistently closest to 100% of their original length, for all larval instars, at both long-term and short-term storage, when compared to the larvae stored in the fridge and at room temperature (Figs. 3 and 4). Despite differences in the change in larval length between storage temperatures being as large as 16.6% (Fig. 3), the overall differences between the trends of change in larval length at different storage temperatures were negligible, yielding non-significant interactions. This demonstrates that *C. vicina* larvae, of any stage, can be stored in 80% ethanol, at any constant temperature ranging from −25°C to +24°C without having to correct for storage temperature.

The duration of preservation had a significant effect on the change in larval length for all instars except post-feeding larvae, where the change in length differed by a maximum average between all storage temperatures of only 2.4% during the total 365 days of storage. These data clearly demonstrate that the degree of change in length to *C. vicina* larvae that have been killed in boiling water and then preserved in 80% ethanol is dependent on the developmental stage of the larvae (Figs. 3 and 4). Second instar larvae were most affected by the amount of time spent in preservation, reducing to 64.8% of the original average length after 365 days. First instar larvae were expected to be most affected during preservation as they have the highest surface area:volume ratio. This large surface area, relative to their volume, would have aided in a high rate of osmosis, resulting in rapid dehydration of the larvae. As a result, first instar larvae reached their minimum size fairly rapidly when compared to the other larval development stages (Fig. 3) but did not reduce in size the most. Second instar larvae contain more water than first instar larvae, due to their larger size, but still have a relatively thinner cuticle, when compared to third instar larvae [9]. It is possible that the thinner cuticle of second instar larvae allowed for the majority of the water within the larvae to diffuse out, resulting in the large reduction in length observed. This explanation is supported by the fact that second instar larvae lost an average of 82.2% of their weight, for all storage temperatures, during preservation after 365 days. However, it is likely that dehydration is not the only factor affecting change in length, and that other processes e.g. dissolving lipids, are at work.

The third instar stage was the only larval stage to show periods of increase in length and weight during preservation. This is particularly true for the feeding third instar stage which had an increased average length after 24 h and after 7 days. These increases appear uncharacteristic in context with the overall trend during short-term (Fig. 4) and long-term (Fig. 3) storage, as the average percentage length of the samples before and after these sample times were noticeably smaller. These fluctuations contribute to an irregular trend in the change in average percentage length over time. This does not add to the complexity of correcting for this change when using these models, but does offer a degree of uncertainty when correcting for this change.

The length of post-feeding larvae remained largely unchanged for the duration of both short-term and long-term storage at all storage temperatures, and differed by a maximum of only 2.4% from the original length. This result was surprising when compared to the responses of feeding third instar larvae and, especially, to those of first and second instar larvae during preservation. However, Tantawi and Greenberg [18], the only other publication on the change in larval length of feeding and post-feeding third instar *C. vicina* during preservation, recorded very similar findings, stating that ‘the percentage of shrinkage was greater for young third instar larvae than for older ones’.

It is difficult to speculate why the change in length of post-feeding third instar larvae was so different to that of feeding third instar larvae. Post-feeding larvae used in this experiment had only just entered the wandering phase and were therefore at their largest (mean = 17.6 mm, s.d. = 1.1 mm, *n* = 60). Their cuticles had expanded to their maximum capacity and may not have facilitated any further expansion due to external factors such as preservation. But, the feeding third instar larvae in this experiment had only recently undergone second ecdysis (mean = 8.6 mm, s.d. = 1.2 mm, *n* = 60) and therefore their cuticles were still very elastic to facilitate growth. This elasticity might have more readily allowed changes in length due to osmotic potential, marked by the unpredictable trend in the change in length of feeding third instar larvae during preservation (Figs. 3 and 4).

Adams and Hall [1] studied the effects of preservation on the change in length of post-feeding *C. vomitoria*, a closely related species, and found an increase in length after the first 12 h of preservation (7.5% of the original length, in comparison to 1.8% increase in our data). Therefore, the degree of change in larval length during preservation is also dependent on species.

### Implication for estimating PMI_min_ using C. vicina larvae

**Fig. 5:**
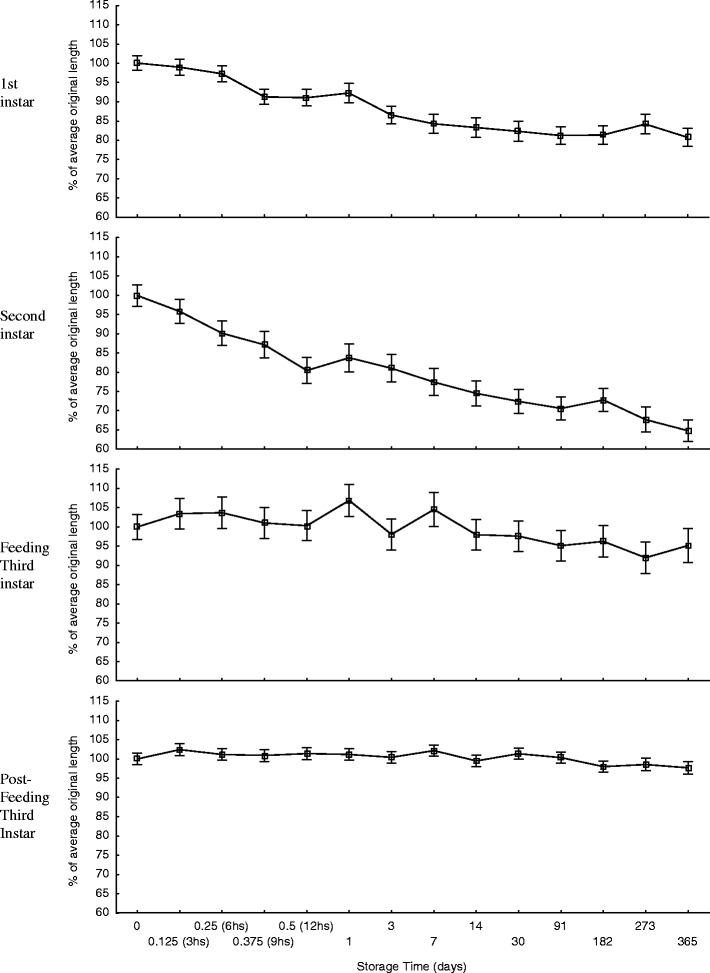
Combined data of the change in larval length for the 365 days of storage at all storage temperatures for first instar, second instar, feeding third instar, and post-feeding third instar larvae. Data are represented by the mean (*unfilled square*, *n* = 60) and corresponding ±95% confidence intervals (*error bars*)

The significant reduction in length during preservation had an amplified significant effect on resultant PMI_min_ calculations (Table 1). The resulting errors in PMI_min_ varied greatly between the different larval instars, ranging from −53.0% for second instar larvae to +5.3% for post-feeding larvae (Table 1). Therefore, to adjust for changes in length of preserved larvae, it is important to know the larval stage and amount of time the larvae have been in a preservative (Figs. 1, 2, 3 and 4). Because storage temperature had no significant effect on the change in larval length during preservation, the length data from the three storage temperatures were pooled and summarised (Fig. 5). The change in length was represented in these figures as a percentage of the original size rather than actual lengths (in mm) to make the model applicable to all larval sizes within each developmental stage. It is not advisable to use this model on larvae that are outside the length range of the sampled larvae used to build this model (size ranges of first, second, feeding third instar and post-feeding larvae was 2.7–3.9 mm, 4.6–8.0 mm, 6.3–12.5 mm, 14.7–19.3 mm, respectively).

Although the percentage error in PMI_min_ is as large as 53.0% for second instar *C. vicina* larvae, this would translate to an error of only 12 h at 20°C, and this error in time would be even smaller at higher temperatures (Table 1). In these instances, an accurate PMI_min_ with a precision of no more than 1 day may be presented with confidence without correcting for this change in length. But, if narrowing down the precision of the estimate is of particular importance to a case, then an effective way to achieve this is to correct for the change in larval length during preservation using the models provided (Fig. 5).

Larval lengths vary within instars (Table 1) and these data show that the smaller larvae, within each instar, produce larger percentage errors in PMI_min_ than larger larvae, even though the proportional change in larval length is regarded as constant for all larvae of the same instar. Therefore, forensic entomologists should be aware of the size of the larvae they are handling relative to the instar of those larvae as this will affect the precision of the estimate if these changes in length are not corrected for.

The length of first and second instar larvae is most affected during preservation and these larval stages should ideally be corrected for when estimating PMI_min_, particularly if a high level of precision is required. The length of feeding third instar larvae is less affected than earlier instars during preservation (75% and 80% of all feeding third instar larvae remained within 5% and 7% of the average original length during short-term and long-term storage, respectively (Figs. 3 and 4)) and corrections for this change need only be taken into account if larvae are stored for 9 months or longer. Finally, no corrections for estimates of PMI_min_ are necessary for post-feeding third instar larvae, as any changes in length of this developmental stage remained within 5% of the original length for the entire duration of this study.

Variation between individuals in changes of size during preservation is inherent and is a source of error (14). For this reason PMI_min_ estimates should be based on a large enough sample population of immature insects rather than a few individuals (*n* < 10). Therefore, if a large enough sample size (*n* > 60, i.e. at least the number of larvae used to build this model) is not available during case work, then we advise caution in using this model to correct for changes in larval size during preservation. However, when using this model to correct for change in length, it is important to include the 95% confidence intervals into any adjustments to the data, along with the variation of any other influencing variables, in any estimated window of PMI_min_.

### Conclusions

- Our data illustrate that larval length is a significantly better measure of age than larval weight when using preserved larvae to estimate time of death.
- Between −25°C and +24°C, storage temperature did not have a significant effect on change in length during preservation.
- The degrees of changes in length of larvae that have been killed in boiling water and then preserved in 80% ethanol are dependent on the developmental stage of the larvae and species.
- Smaller larvae, relative to the size range of their instar, have a greater effect on the error of resulting PMI_min_ estimates than larger larvae.
